# Validation of scoring systems for the prediction of complicated appendicitis in adults using clinical and computed tomographic findings

**DOI:** 10.1186/s13244-023-01540-4

**Published:** 2023-11-16

**Authors:** Rathachai Kaewlai, Sasima Tongsai, Wanwarang Teerasamit, Dhanawin Wongsaengchan, Napakadol Noppakunsomboon, Pramuk Khamman, Anchisa Chatkaewpaisal, Piyaporn Apisarnthanarak

**Affiliations:** 1 Department of Radiology, Faculty of Medicine Siriraj Hospital, Mahidol University, 2 Wanglang Rd, Bangkok Noi,, Bangkok, 10700 Thailand; 2 Department of Research, Faculty of Medicine Siriraj Hospital, Mahidol University, 2 Wanglang Rd, Bangkok Noi, Bangkok, 10700 Thailand; 3 Department of Surgery, Faculty of Medicine Siriraj Hospital, Mahidol University, 2 Wanglang Rd, Bangkok Noi, Bangkok, 10700 Thailand; 4 Department of Anatomy, Faculty of Medicine Siriraj Hospital, Mahidol University, 2 Wanglang Rd, Bangkok Noi, Bangkok, 10700 Thailand

**Keywords:** Adult, Appendicitis, ROC Curve, Scoring system, Multidetector computed tomography

## Abstract

**Objectives:**

The study aimed to evaluate scoring systems for predicting complicated appendicitis in adults diagnosed with acute appendicitis on computed tomography.

**Methods:**

Three hundred twenty-five consecutive adult patients (mean age 51.9 ± 19.6 years, 212 women) diagnosed with acute appendicitis on computed tomography were retrospectively included. Clinical and imaging findings were compared between patients with and without complicated appendicitis, and independent associations were identified. As C-reactive protein was not available for most patients, 5 out of 8 scoring systems were modified. They, and a newly proposed system, were compared via area under the receiver operating characteristics (ROC) curve (AUC), Additionally, the latter was internally validated. Pairwise comparison was performed, and diagnostic performance of these scoring systems was obtained.

**Results:**

One hundred twenty-seven patients (36.8%) had complicated appendicitis. Significant independent associations were found between complicated appendicitis and duration of symptoms > 12 h, appendicolith, periappendiceal fat stranding, periappendiceal fluid, and extraluminal air (*p* values < 0.001 to 0.037; AUCs of 0.824–0.829). AUCs of 9 scoring systems ranged from 0.692 to 0.831. Of these, modified Atema, Kim HY, and proposed scores had similarly high and non-significantly different AUCs (0.793–0.831) on pairwise comparison. Their sensitivities, specificities, and accuracies were 73.0–90.6%, 48.5–70.6%, and 64.3–72.3%, respectively. Internal validity test demonstrated high AUCs (0.826–0.844) with one of the proposed scores using odds ratio having 100% sensitivity and 100% negative predictive value.

**Conclusions:**

Few scoring systems, including proposed ones, had high AUCs, sensitivity, and reasonable specificities, which could potentially aid in safely selecting adult patients with acute appendicitis for nonoperative management.

**Critical relevance statement:**

The study suggests few scoring systems for predicting complicated appendicitis with high AUCs and reasonable sensitivities, potentially aiding in selecting patients for nonoperative management.

**Key points:**

• The study evaluated existing and proposed new scoring systems to predict complicated appendicitis in adults with acute appendicitis on computed tomography.

• Several factors were found to be significantly associated with complicated appendicitis, including duration of symptoms, appendicolith, periappendiceal fat stranding, periappendiceal fluid, and extraluminal air.

• The modified Atema, Kim HY, and newly proposed scoring systems performed well, potentially aiding in nonoperative management selection.

**Graphical Abstract:**

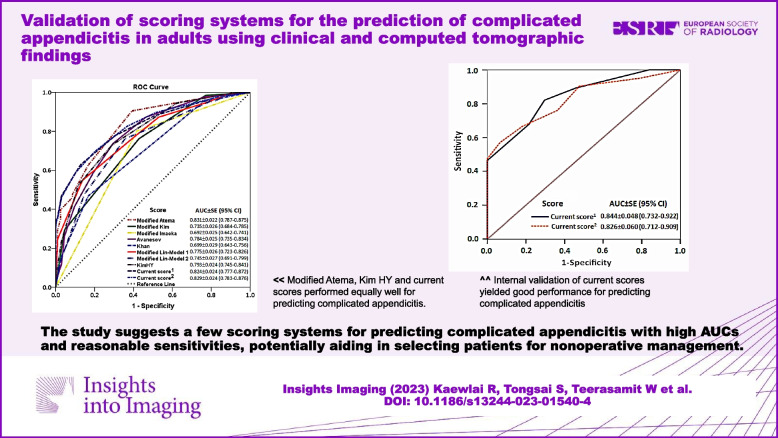

**Supplementary Information:**

The online version contains supplementary material available at 10.1186/s13244-023-01540-4.

## Introduction

Acute appendicitis is a common emergency condition in adults, which can result in severe complications if not managed appropriately. Complicated appendicitis can lead to perforation, abscess formation, peritonitis, and sepsis and require urgent surgical intervention [1]. Conversely, uncomplicated appendicitis can be treated with either appendectomy or nonoperative management with the use of antibiotics [2]. Nonoperative management is a viable option for selected patients with uncomplicated appendicitis, particularly those who are at increased risk for surgical complications or have a preference for a nonsurgical approach. Patient selection is, therefore, crucial in identifying those with uncomplicated appendicitis and avoiding directing complicated cases to a nonsurgical approach. The guideline issued by the World Society of Emergency Surgery emphasizes the importance of patient selection in the management of acute appendicitis [1].

Clinical scoring systems have been developed to aid in diagnosing appendicitis, such as the Alvarado score, Appendicitis Inflammatory Response score, and Adult Appendicitis Score. However, these scores have limited ability to differentiate between uncomplicated and complicated appendicitis [3, 4]. Several scoring systems have been proposed to aid in identifying complicated appendicitis, with varying methods and success [5–11]. However, only a few studies [10, 12, 13] have externally validated their performance.

Our investigation aimed to evaluate the performance of existing scoring systems for predicting complicated appendicitis in adults diagnosed with acute appendicitis on computed tomography and compare them to a newly proposed system.

## Methods

### Study design and patient selection

**Fig. 1:**
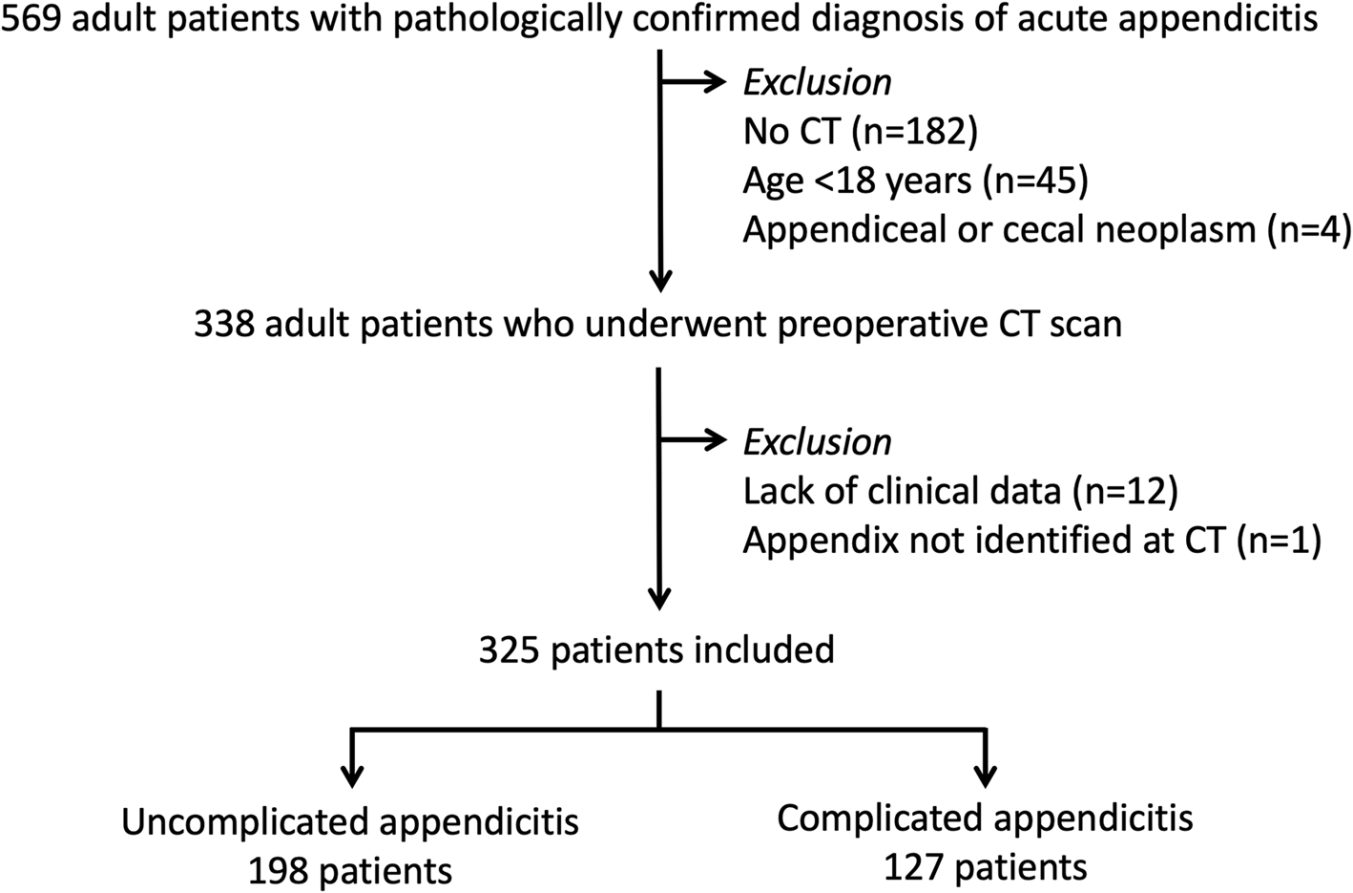
Flow chart of patient inclusion

This investigation was conducted at a 2200-bed urban academic hospital. It was approved by the Institutional Review Board (protocol No. 136/2566(IRB2)). Informed consent was not required due to the retrospective nature and minimal risk involved. Figure 1 provides a flow chart of patient inclusion. The study identified eligible patients by searching the pathological database for a diagnosis of appendicitis among all consecutive adult patients aged 18 years or older from October 2016 to March 2021. Patients who had undergone abdominopelvic CT prior to appendectomy, regardless of the timing of appendectomy relative to the diagnosis of acute appendicitis, were included. Only the first CT examination indicating a clinical suspicion of acute appendicitis was included if there were multiple CT exams. Patients with incomplete clinical data (*n* = 12) and an appendix not identified at CT (*n* = 1) were excluded. The investigation ultimately included 325 patients (Table 1). Note that 201 of these patients have been described in our previous investigation [14]. Among the 325 patients, 321 initially underwent a CT scan as their primary imaging modality, while the remaining individuals had an initial ultrasound examination.

**Table 1:** Patient characteristics and comparison between uncomplicated and complicated appendicitis (*n* = 325)

Factors	All patients (*n* = 325)	Uncomplicated (*n* = 198)	Complicated (*n* = 127)	*p* values
**Demographics**				
Age (years; mean ± SD)	51.9 (19.6)	48.6 (19.0)	57.0 (19.6)	< *0.001*
Age group (years) ≥ 45	196 (60.3%)	106 (53.5%)	90 (70.9%)	*0.003*
Age group (years) ≥ 52	169 (52.0%)	89 (44.9%)	80 (63.0%)	*0.002*
Age group (years; *n*, %)				*0.020*
< 40	105 (32.3%)	75 (37.9%)	30 (23.6%)	
40–59	88 (27.1%)	52 (26.3%)	36 (28.4%)	
≥ 60	132 (40.6%)	71 (35.9%)	61 (48.0%)	
Female (*n*, %)	212 (65.2%)	130 (65.7%)	82 (64.6%)	0.935
Body mass index (kg/m^2^; mean ± SD)	24.0 (4.7)	24.0 (4.8)	24.0 (4.7)	0.966
**Durations (h; median, range**^**a**^**)**				
Duration of symptoms	24 (2–480)	24 (2–240)	36 (3–480)	< *0.001*
Duration of symptoms (h) ≥ 48	105 (32.3%)	42 (21.2%)	63 (49.6%)	< *0.001*
From arrival to CT	5.2 (0.2–82.1)	5.1 (0.2–27.5)	5.3 (0.5–82.1)	0.154
From CT to surgery	4.0 (0.3–74.1)	4.1 (0.4–74.1)	3.5 (0.3–47.1)	0.264
From arrival to surgery	9.6 (3.0–87.6)	9.5 (3.0–87.6)	9.8 (3.4–52.5)	0.364
From arrival to antibiotics	6.5 (0–29.4)	6.5 (0.5–29.4)	6.5 (0–20.4)	0.393
**Symptoms and signs**				
Right lower quadrant pain (*n*; %)	314 (96.6%)	193 (97.5%)	121 (95.3%)	0.350
Body temperature (°C; mean ± SD)	37.3 (0.8)	37.2 (0.7)	37.5 (0.9)	*0.001*
Body temperature group (°C)				*0.001*
≤ 37.0	166 (51.2%)	113 (57.4%)	53 (41.7%)	
37.1–37.9	105 (32.4%)	63 (32.0%)	42 (33.1%)	
≥ 38	53 (16.4%)	21 (10.7%)	32 (25.2%)	
Body temperature group (°C) ≥ 37.4	106 (32.6%)	60 (30.3%)	46 (36.2%)	0.323
Body temperature group (°C) ≥ 37.5	97 (29.8%)	52 (26.3%)	45 (35.4%)	0.101
Rebound tenderness (*n*; %)	162 (49.8%)	95 (48.0%)	67 (52.8%)	0.468
Migratory pain (*n*; %)	143 (44.0%)	101 (51.0%)	42 (33.1%)	*0.002*
Anorexia (*n*; %)	159 (48.9%)	85 (42.9%)	74 (58.3%)	*0.010*
Nausea and vomiting (*n*; %)	190 (58.5%)	110 (55.6%)	80 (63.0%)	0.225
**Laboratory values**				
White blood cell count (× 10^9^ cells/L; mean ± SD)	13.2 (3.0–29.2)	12.8 (3.0–24.7)	13.3 (3.9–29.2)	0.328
White blood cell count group > 13 (× 10^9^ cells/L)	169 (52.0%)	98 (49.5%)	71 (55.9%)	0.310
Neutrophil count (%; median, range)	82.6 (21.0–96.7)	81.2 (21.0–96.7)	85.2 (28.6–95.2)	< *0.001*
Neutrophil count ≥ 81% (n, %)	200 (61.9%)	109 (55.3%)	91 (72.2%)	*0.003*
Absolute neutrophil count (× 10^9^ cells/L)	10.6 (1.5–26.6)	10.4 (1.5–22)	11.3 (1.9–26.6)	*0.049*
Leukocyte count (%; median, range)	11 (1.0–94.6)	11.4 (1.0–94.6)	10.0 (1.6–51.0)	*0.003*
Neutrophil-to-leukocyte ratio (median, range)	7.5 (0.04–71.7)	7.1 (0.04–71.7)	8.2 (0.8–59.6)	*0.007*
Neutrophil-to-leukocyte ratio > 10 (*n*, %)	104 (32.2%)	59 (29.9%)	45 (35.7%)	0.337
**Alvarado score (median, range)**	7 (1–10)	7 (2–10)	7 (1–10)	0.275
**CT findings**				
Appendix diameter (mm; mean ± SD)	12.0 (2.9)	11.2 (6–21.1)	13 (8.6–26.8)	< *0.001*
Appendix diameter > 10 mm	245 (75.4%)	134 (67.7%)	111 (87.4%)	< *0.001*
Appendix diameter ≥ 14 mm	77 (23.7%)	34 (17.2%)	43 (33.9%)	< *0.001*
Appendicolith (*n*, %)	134 (41.2%)	58 (29.3%)	76 (59.8%)	< *0.001*
Obstructive appendicolith (*n*, % of appendicolith) (*n* = 136)	88 (64.7%)	37 (61.7%)	51 (67.1%)	0.632
Number of appendicolith (*n*; median, range)	0 (0–8)	0 (0–6)	1 (0–8)	0.7178
Location of appendicolith (*n*, %) (*n* = 134)				0.399
Proximal	86 (64.2%)	36 (62.1%)	50 (65.8%)	
Mid	28 (20.9%)	15 (25.9%)	13 (17.1%)	
Distal	20 (14.9%)	7 (12.1%)	13 (17.1%)	
Contrast enhancement wall defect (*n*, %)	148 (45.5%)	66 (33.3%)	82 (64.6%)	< 0.001
Periappendiceal fat stranding (*n*, %)	152 (46.8%)	62 (31.3%)	90 (70.9%)	< *0.001*
Periappendiceal fluid (*n*, %)	138 (42.5%)	49 (24.7%)	89 (70.1%)	< *0.001*
Abscess (*n*, %)	42 (12.9%)	22 (11.1%)	20 (15.7%)	0.295
Ascites (*n*, %)	110 (33.8%)	51 (25.8%)	59 (46.5%)	< *0.001*
Extraluminal air (*n*, %)	47 (14.5%)	2 (1.0%)	45 (35.4%)	< *0.001*
**Treatment**				
Appendectomy at initial admission (*n*, %)	316 (97.2%)	192 (97.0%)	124 (97.6%)	1.000
Length of stay for initial admission (days; median, range)	3 (1, 44)	2 (1, 13)	4 (1, 44)	< *0.001*

^a^Unless specified separately

### Image acquisition, reinterpretation, and definitions

One of the three multidetector CT scanners was used to conduct CT exams. With the exception of one scan, all exams were performed with intravenous administration of nonionic iodinated contrast medium, at a volume of 1.5–2.0 mL/kg (equivalent to approximately 80–100 mL) at a rate of 2–3 mL/s. The exams covered the area from either the upper border of the diaphragms or the upper pole of the kidneys to the ischial tuberosities. For each scan, an unenhanced phase was followed by a portovenous phase (approximately 80 s after contrast administration) with an axial slice thickness of 1.25 mm. All images were then transferred to Picture Archiving and Communication Systems (PACS) for viewing.

Two fellowship-trained radiologists, specialized in abdominal imaging and emergency imaging with 20 years of experience each, independently re-reviewed all CT scans. They were informed of the patient’s age, sex, and diagnosis of acute appendicitis, but remained unaware of other data. The images were analyzed on standard PACS workstations using Synapse (FujiFilm Inc.). Each radiologist provided their own interpretation of the CT findings based on definitions described in Supplementary Material 1 and previously [14]. Discrepancies between the two radiologists were resolved by an abdominal radiologist with 24 years of experience for the 201 previously reported cases, while the rest were resolved by consensus.

### Reference standards

The diagnosis of acute appendicitis was confirmed through histopathological analysis. Cases of complicated appendicitis included those with gangrene or perforation. The diagnosis of gangrene was based on histopathology, while perforation was documented either through histopathology or surgical operative findings.

### Scoring systems validated

Eight scoring systems were selected for validation due to their inclusion of both clinical features and CT findings in their scores [5–11]. Details of these scores are provided in Supplementary Material 2. Of these, 5 included serum C-reactive protein in their scores [5, 6, 10], which was documented in only 7 of our patient cohort. Therefore, this laboratory value was removed from the scores. The weighting of included factors remained but the appropriate cutoff values for all scores were reselected during statistical analysis.

### Statistical analysis

Qualitative and quantitative information were analyzed using descriptive statistics. Categorical variables were presented in terms of numbers or percentages while continuous data were reported as mean (standard deviation) or median (range) depending on whether the data had normal or skewed data distribution.

To compare the difference between the two groups (uncomplicated vs. complicated appendicitis), inferential statistics were used. The Pearson chi-square or Fisher’s exact test was used for categorical variables, while the independent *t*-test or the Mann–Whitney *U* test was used for continuous variables having means or medians, respectively. Binary logistic regression was used for univariable and multivariable analyses to determine the odds ratio (OR) and coefficients for independent predictors of complicated appendicitis. Odds ratio with corresponding 95% confidence interval (95% CI) were used to identify the strength and direction of their association. The selection of factors into the multivariable model was based on a *P* value of less than 0.1 in the univariable model. In order to prioritize patient safety, we placed a high emphasis on sensitivity to diagnose complicated appendicitis. This approach enables the safe practice of recommending appendectomy for patients with uncomplicated appendicitis, rather than resorting to nonoperative management for those with complicated appendicitis.

The diagnostic performance of the scoring systems in differentiating between uncomplicated and complicated appendicitis was determined using two-by-two tables to calculate metrics such as sensitivity, specificity, positive likelihood ratio, negative likelihood ratio, positive predictive value, negative predictive value, and accuracy. The ROC curves of these scoring systems were compared through pairwise comparison. These analyses were conducted using the Statistical Package for Social Sciences (SPSS, version 23, IBM), with a significance of 0.05.

The discrimination of the scoring systems describes the ability to give different predictions for complicated and uncomplicated appendicitis. The area under the ROC curve (AUC) with the corresponding 95% confidence interval (95% CI) was considered a summary measure for discrimination. The internal validation of the model was carried out by split-sample estimation and validation, in which the entire sample was randomly divided into two subsets, one used exclusively for model estimation ("training") and another used for validation ("testing"). Data were randomly divided with a split-sample approach, with 80% of the data allocated for training the model and 20% for internal validation using the R program (R Core Team (2022). R: A language and environment for statistical computing. R Foundation for Statistical Computing, Vienna, Austria. URL https://www.R-project.org/).

## Results

### Baseline characteristics of patients (Table 1)

The mean age of the patients was 51.9 ± 19.6 years. Most of them (60.3%) belonged to the age group of ≥ 45 years, with female predominance (65.2%). They presented to the hospital with a median duration of symptoms of 24 h (range, 2–480) and a median Alvarado score of 7 (range, 1–10). On CT, the mean appendix diameter was 12.0 ± 2.9 mm, and 41.2% of patients had an appendicolith. Periappendiceal fat stranding, periappendiceal fluid, ascites, and extraluminal air were present in 46.8%, 42.5%, 33.8%, and 14.5% of cases, respectively. One hundred twenty-seven patients (39.1%) had complicated appendicitis. Almost all patients (97.2%) had appendectomy at the initial admission of appendicitis. The median length of stay was 3 days (range, 1–44).

### Predictive factors of complicated appendicitis (Tables 2 and 3)

**Table 2:** Univariable and multivariable analyses of predictive factors of complicated appendicitis

**Factors**	**Univariable model**		**Multivariable model**	
	**Unadjusted OR (95% CI)**	***p*** **-value**	**Adjusted OR (95% CI)**	***p*** **-value**
Duration of symptoms > 12 h	3.466 (1.968–6.103)	< 0.001	2.403 (1.246–4.636)	*0.009*
Appendicolith	3.597 (2.251–5.747)	< 0.001	1.855 (1.039–3.312)	*0.037*
Periappendiceal fat stranding	5.336 (3.280–8.579)	< 0.001	2.850 (1.592–5.104)	< *0.001*
Periappendiceal fluid	7.122 (4.326–11.724)	< 0.001	2.721 (1.511–4.899)	*0.001*
Extraluminal air	53.780 (12.747–226.908)	< 0.001	15.407 (3.421–69.382)	< *0.001*

*OR*, odds ratio. The independent variables with *p*-value < 0.10 in simple binary logistic regression model and without multicollinearity were included in multivariable analysis

**Table 3:** Weighted score for each factor in the risk prediction of complicated appendicitis

Factors	Category	Adjusted OR	Points	Coefficient	Points
Duration of symptoms > 12 h	≤ 12	Reference	0	Reference	0
	> 12	2.403	1	0.877	1
Appendicolith	Yes	1.855	1	0.618	1
Periappendiceal fat stranding	Yes	2.850	2	1.047	2
Periappendiceal fluid	Yes	2.721	1	1.001	2
Extraluminal air	Yes	15.407	8	2.735	4

Univariable analysis identified multiple clinical, laboratory, and imaging factors that are significantly associated with complicated appendicitis. After multivariable analysis, five factors remained statistically significant: duration of symptoms > 12 h, appendicolith, periappendiceal fat stranding, periappendiceal fluid, and extraluminal air. Their *p* values ranged from < 0.001 to 0.037. The odds ratios and coefficients were weighted for each factor to identify the risk prediction of complicated appendicitis as shown in Table 3.

### Comparison of 9 scoring systems (Table 4)

**Table 4:** Diagnostic performance of 10 scores

	**Modified Atema score**	**Modified Kim score**	**Modified Imaoka score**	**Avanesov score**	**Khan score**	**Modified Lin Model 1 score**	**Modified Lin Model 2 score**	**KimHY score**	**Current score** ^**a**^	**Current score** ^**b**^
Cutoff values	≥ 5	≥ 1	≥ 1	≥ 2	≥ 2	≥ 4	≥ 4	≥ 3	≥ 2	≥ 2
True positive	115	125	101	111	96	111	106	92	112	114
False positive	78	153	83	92	103	105	107	58	101	102
False negative	12	2	26	16	31	16	20	34	15	13
True negative	120	45	115	105	95	93	90	139	97	96
Sensitivity (%)	90.6 (84.1–95.0)	98.4 (94.4–99.8)	79.5 (71.5–81.2)	87.4 (80.3–92.6)	75.6 (67.2–82.8)	87.4 (80.3–92.6)	84.1 (76.6–90.0)	73.0 (64.4–80.5)	88.2 (81.3–93.2)	89.8 (82.1–94.4)
Specificity (%)	60.6 (53.4–67.5)	22.7 (17.1–29.2)	58.1 (50.9–65.0)	52.3 (46.1–60.4)	48.0 (40.8–55.2)	47.0 (39.9–54.2)	45.7 (38.6–52.9)	70.6 (63.7–76.8)	49.0 (41.8–56.2)	48.5 (41.3–55.7)
Positive likelihood ratio	2.30 (1.92–2.76)	1.27 (1.18–1.38)	1.90 (1.58–2.29)	1.87 (1.59–2.20)	1.45 (1.23–1.72)	1.65 (1.42–1.91)	1.55 (1.34–1.80)	2.48 (1.95–3.16)	1.73 (1.49–2.01)	1.74 (1.50–2.02)
Negative likelihood ratio	0.16 (0.09–0.27)	0.07 (0.02–0.28)	0.35 (0.25–0.51)	0.24 (0.15–0.38)	0.51 (0.36–0.71)	0.27 (0.17–0.43)	0.35 (0.23–0.53)	0.38 (0.28–0.52)	0.24 (0.15–0.40)	0.21 (0.12–0.36)
Positive predictive value (%)	59.6 (55.2–63.9)	45.0 (43.0–46.9)	54.9 (50.3–59.4)	54.7 (50.6–58.7)	48.2 (44.1–52.4)	51.4 (47.7–55.0)	49.8 (46.1–53.5)	61.3 (55.5–66.9)	52.6 (48.8–56.3)	52.8 (49.1–56.4)
Negative predictive value (%)	90.9 (85.2–94.6)	95.8 (84.8–98.9)	81.6 (75.5–86.4)	86.8 (80.3–91.4)	75.4 (68.6–81.1)	85.3 (78.2–90.4)	81.8 (74.5–87.4)	80.3 (75.2–84.7)	86.6 (79.8–91.4)	88.1 (81.2–92.6)
Accuracy (%)	72.3 (67.1–77.1)	52.3 (46.7–57.8)	66.5 (61.0–71.6)	66.7 (61.2–71.8)	58.8 (53.2–64.2)	62.8 (57.3–68.0)	60.7 (55.1–66.0)	71.5 (66.3–76.4)	64.3 (58.8–69.5)	64.6 (59.2–69.8)

^a^Using odds ratio

^b^Using coefficient

**Fig. 2:**
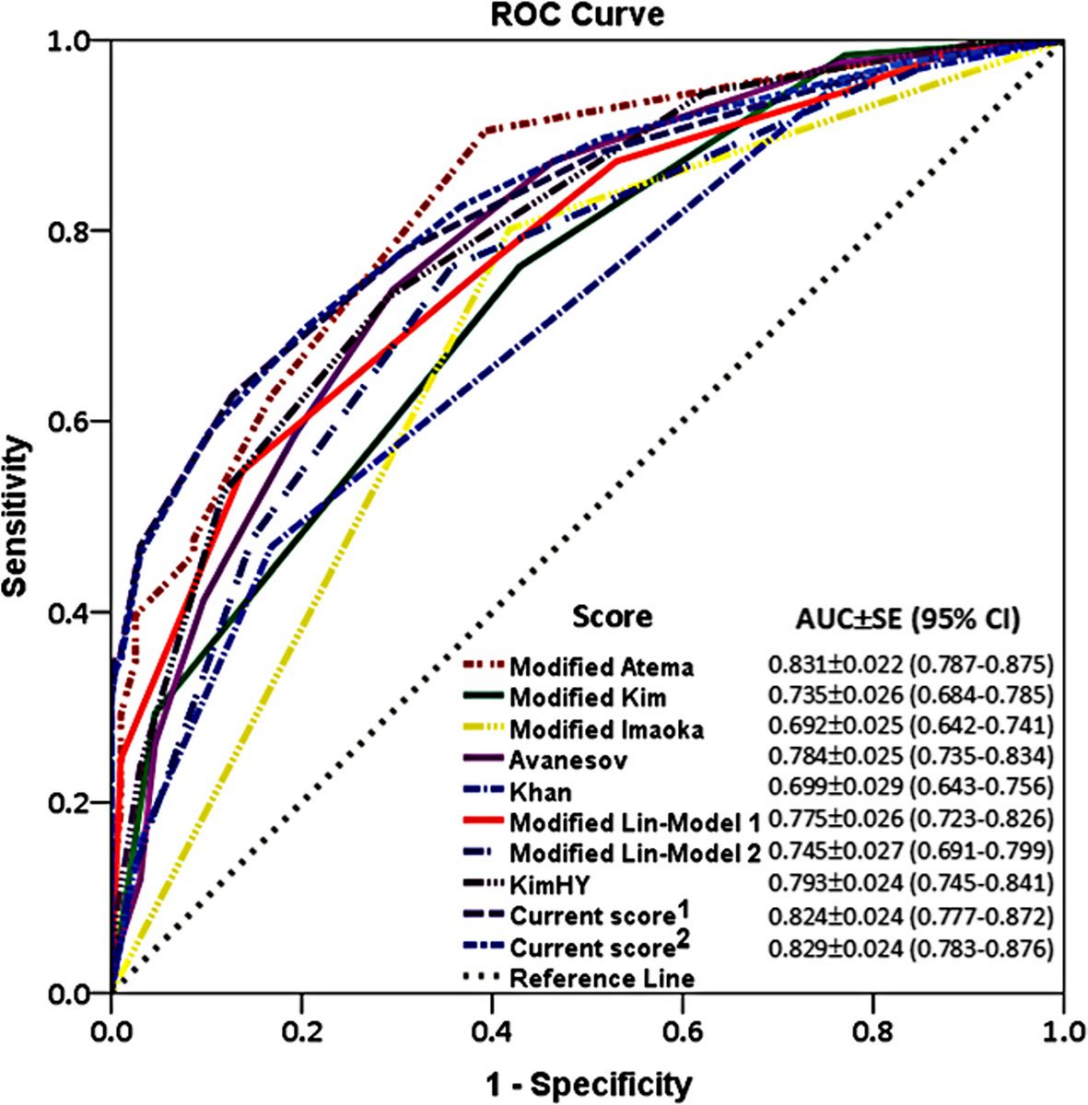
Comparison of ROC curves of 8 scoring systems and current scores

The Atema, Kim, Imaoka, and Lin (models 1 and 2) scores were modified to exclude C-reactive protein, with their respective cutoff values selected at ≥ 5, ≥ 1, ≥ 1, ≥ 4, and ≥ 4, respectively. The cutoff value of the Avanesov score was reduced from ≥ 4 in the original description to ≥ 2 in our analysis. The Khan and Kim HY scores retained their original cutoff values of ≥ 2 and ≥ 3, respectively. Their AUCs are provided in Fig. 2. The scores based on our multivariable analysis assigned different points to each predictive factor. For both, when a value of ≥ 2 was used as a cutoff, the scores (based on odds ratios or coefficients) demonstrated a sensitivity, specificity, and accuracy of 88.2–89.8%, 48.5–49.0%, and 64.3–64.6%, respectively. The one that utilized the coefficients had slightly better sensitivity and accuracy, but slightly less specificity. Pairwise comparison of these ten scores (Table 5) revealed no significant difference between the modified Atema, Kim HY, and our (identified as “current”) proposed scores (*p* = 0.110–0.901).

**Table 5:** Pairwise comparison of area under the ROC curves of 10 scores

	**Modified Atema score ≥ 5**	**Modified Kim score ≥ 1**	**Modified Imaoka score ≥ 1**	**Avanesov score ≥ 2**	**Khan score ≥ 2**	**Modified Lin Model 1 score ≥ 4**	**Modified Lin Model 2 score ≥ 4**	**KimHY score ≥ 3**	**Current score**^**a**^** ≥ 2**	**Current score**^**b**^** ≥ 2**
Modified Atema score ≥ 5	1	0.096 *(0.043 to 0.149)*	0.140 *(0.096 to 0.184)*	0.047 *(0.012 to 0.082)*	0.132 *(0.081 to 0.183)*	0.057 *(0.011 to 0.102)*	0.087 *(0.035 to 0.138)*	0.038 (− 0.009 to 0.085)	0.007 (− 0.030 to 0.043)	0.002 (− 0.033 to 0.037)
Modified Kim score ≥ 1	< *0.001*	1	0.043 (− 0.013 to 0.099)	− 0.049 (− 0.104 to 0.005)	0.035 (− 0.035 to 0.106)	− 0.040 (− 0.076 to − *0.033)*	− 0.010 (− 0.047 to 0.027)	− 0.058 *(*− *0.101 to* − *0.015)*	− 0.090 *(*− *0.135 to* − *0.044*)	− 0.094 *(*− *0.139 to* − *0.050)*
Modified Imaoka score ≥ 1	< *0.001*	0.130	1	− 0.093 *(*− *0.136 to* − *0.049)*	− 0.008 (− 0.081 to 0.065)	− 0.083 *(*− *0.139 to* − *0.027)*	− 0.053 (− 0.111 to 0.005)	− 0.101 *(*− *0.159 to* − *0.043)*	− 0.133 *(*− *0.183 to* − *0.082)*	− 0.137 *(*− *0.183 to* − *0.092)*
Avanesov score ≥ 2	*0.008*	0.077	< *0.001*	1	0.085 *(0.027 to 0.142)*	0.010 (− 0.043 to 0.063)	0.040 (− 0.017 to 0.096)	− 0.009 (− 0.056 to 0.039)	− 0.040 (− 0.084 to 0.004)	− 0.045 *(*− *0.084 to* − *0.005)*
Khan score ≥ 2	< *0.001*	0.327	0.834	*0.004*	1	− 0.075 *(*− *0.139 to* − *0.012)*	− 0.045 (− 0.116 to 0.025)	− 0.093 *(*− *0.160 to* − *0.027)*	− 0.125 *(*− *0.186 to* − *0.065)*	− 0.130 *(*− *0.191 to* − *0.068)*
Modified Lin Model 1 score ≥ 4	*0.015*	*0.033*	*0.004*	0.723	*0.021*	1	0.030 *(0.005 to 0.054)*	− 0.018 (− 0.063 to 0.027)	− 0.050 *(*− *0.078 to* − *0.022)*	− 0.054 *(*− *0.085 to* − *0.024)*
Modified Lin Model 2 score ≥ 4	*0.001*	0.602	0.071	0.170	0.209	*0.017*	1	− 0.048 *(*− *0.095 to* − *0.001)*	− 0.080 *(*− *0.118 to* − *0.041)*	− 0.084* (*− *0.124 to* − *0.045)*
KimHY score ≥ 3	0.110	*0.009*	*0.001*	0.723	*0.006*	0.425	*0.044*	1	− 0.032 (− 0.075 to 0.011)	− 0.036 (− 0.080 to 0.007;)
Current score^1^ ≥ 2	0.714	< *0.001*	< *0.001*	0.073	< *0.001*	< *0.001*	< *0.001*	0.150	1	− 0.005 (− 0.015 to 0.006)
Current score^2^ ≥ 2	0.901	< *0.001*	< *0.001*	*0.026*	< *0.001*	< *0.001*	< *0.001*	0.103	0.375	1

Paired-sample area differences under the ROC curves with corresponding 95% confidence intervals were presented in the upper diagonal, and the *p* values between the pairs were presented in the lower diagonal. Values in italics indicate statistical significance

^a^Using odds ratio

^b^Using coefficient

### Internal validation of current scores (Fig. 3, Supplementary Material 3)

**Fig. 3:**
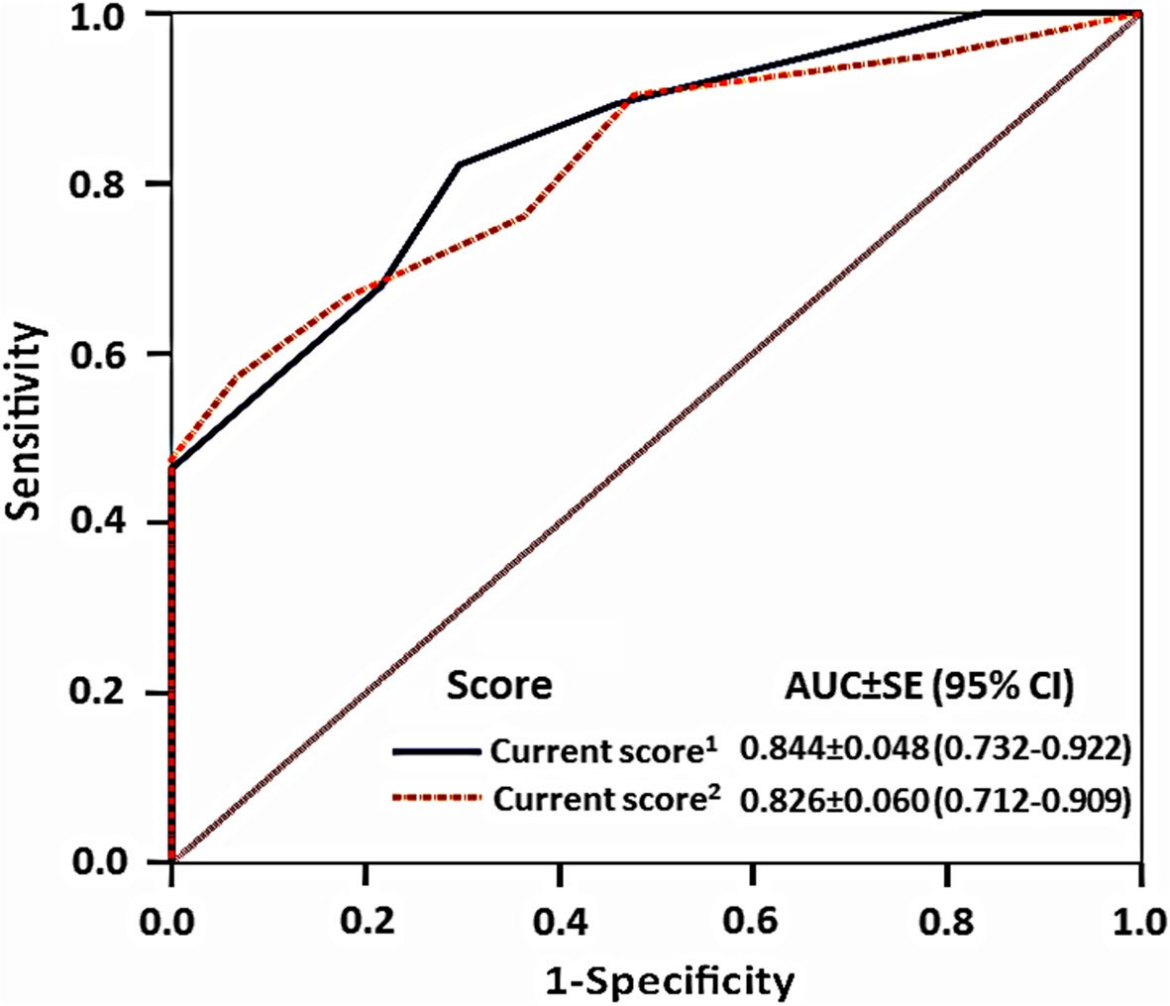
ROC curves of current scoring systems based on internal validation

With a split-model method, 260 cases were allocated for training the model and 65 cases for internal validation of our proposed scores. The scores derived from odds ratios and coefficients both achieved high AUCs (0.826–0.844) with the score using the odds ratio showing a sensitivity and a negative predictive value of 100%, and specificity of 46.4% in predicting complicated appendicitis.

## Discussion

Our investigation identified factors independently predictive of complicated appendicitis that are crucial to consider in the era of potential nonoperative management of acute appendicitis. We validated the diagnostic performance of 8 existing scoring systems and proposed a new scoring system to predict complicated appendicitis without the need for serum C-reactive protein. Of these, modified Atema, Kim HY, and our proposed scores showed similarly high AUCs with reasonably high sensitivities and modest specificities in the identification of complicated appendicitis.

Since 2015, multiple scoring systems have been proposed to identify appendicitis with complications, utilizing clinical-only [15–18], imaging-only [19], or both clinical and imaging data [5–11]. In this study, we validated eight systems that utilized both clinical features and CT findings as these scores generally performed better than those utilized only clinical or CT features. Previous investigations have validated these models using a traditional statistical methodology [10, 12, 13] and artificial neural network [20]. Fujiwara et al. [13], Lin et al. [10], and Geerdink et al. [12] used 203 to 678 patients (52 to 175 with complicated appendicitis) for validation. In another study by Lin et al. [20], datasets of 592 patients were split for training of and validated by artificial neural network.

The Atema score [5] was introduced in 2015, with an original sensitivity of 97% and specificity of 46% in the differentiation of complicated from uncomplicated appendicitis. The scores demonstrated sensitivities from 64 to 90% and specificities from 51 to 95% in subsequent studies [10, 12, 13, 20]. Our investigation found that even with C-reactive protein excluded from the equation and a cutoff value reduced to ≥ 5, the Atema score still had the best performance with high AUC (0.831; 95% CI 0.787–0.875) and sensitivity (91%; 95% CI 84–95%). However, its specificity was only 61% (95% CI 53–68%).

Another scoring system that demonstrated promising results in our investigation was the Kim HY score [11]. In its original description, this score had an AUC of 0.81, a sensitivity of 93%, and a specificity of 28%. However, subsequent validations reported higher AUCs ranging from 0.84 to 0.92 and specificities between 88 and 100%, but lower sensitivities at 64% [10, 20]. Our study showed a balanced sensitivity and specificity at 73% (95% CI 64–81%), and 71% (95% CI 64–77%), respectively, indicating its potential usefulness. Other validated scoring systems showed varying results, with some demonstrating high specificity (Kim TH, Lin Model 2 scores), and others exhibiting variable performance (Imaoka, Avanesov, Khan, Lin Model 1 scores) [10, 13, 20].

Our proposed scoring system, when validated internally, the score that used odds ratio demonstrated 100% sensitivity and 100% negative predictive value, allowing it to avoid misclassification of complicated appendicitis, albeit at a moderate specificity. It overcame the modified Atema score in terms of less complexity as it consisted of only 5 factors for calculation, did not require C-reactive protein, and accumulated fewer total points.

The performance of other scoring systems in our evaluation was suboptimal. Specifically, the Khan score exhibited a lower AUC of 0.699 (95% CI 0.643–0.756), alongside moderate sensitivity (76%; 95% CI 67–83%) and specificity (48%; 95% CI 41–55%). Similarly, the modified Imaoka score demonstrated a lower AUC of 0.692 (95% CI 0.642–0.741), with moderate sensitivity (80%; 95% CI 72–81%) and specificity (58%; 95% CI 51–65%). Both of these were validated by Lin et al. [10], who reported similar diagnostic performance for predicting complicated appendicitis. Additionally, the Imaoka score had been validated by other studies [13, 20], revealing inconsistent diagnostic performance. For the modified Kim score, it exhibited very high sensitivity (98%; 95% CI 94–100%) but low specificity (23%; 95% CI 17–29%), limiting its utility. Notably, our results diverged significantly from the validation performed by Lin et al. [10, 20], who reported the original score as having much lower sensitivity but higher specificity.

When comparing the elements within the scoring systems that exhibited optimal vs. suboptimal performance, the factors contributing the most to enhanced performance were CT findings. Notably, the presence of extraluminal air, which was found in the modified Atema, Kim HY, and our proposed scores but absent in the modified Imaoka, Kim, or Khan scores, played a significant role. Additionally, the presence of appendicolith, which was included in the modified Atema and our proposed score but excluded from the modified Imaoka and Kim scores, also contributed to improved performance.

While our investigation provided a detailed evaluation of the performance of existing scoring systems, there are several limitations that need to be acknowledged. Firstly, our study was retrospective and conducted in a single center with a small sample size. As appendectomy remained the standard of care for acute appendicitis in our hospital, we were unable to evaluate the success rate of nonoperative management fully. However, our approach allowed us to use pathological results as a standard reference for the diagnosis of complicated appendicitis. Secondly, the absence of C-reactive protein data in most patients prevented us from validating some scores in full. However, this allowed us to test the scores without C-reactive protein and demonstrated that the modified Atema score still performed well. Thirdly, we designed our endpoint to prioritize high sensitivity to detect complicated appendicitis, rather than balancing the sensitivity and specificity. This approach ensured patient safety by avoiding sending complicated appendicitis for nonoperative management. Fourthly, we did not validate scores that utilized only clinical factors [16–18] as they were not our target population. Cross-sectional imaging is necessary for safe selection of nonoperative management in this condition even in young individuals [3, 21]. The scores proposed by Mahankali et al. [19] which utilized purely CT findings were not validated in our study due to incomplete data. Additionally, we believe that some data points including grading of periappendicial fat stranding [10] may pose a challenge in terms of real-world applicability as they were subjective.

In conclusion, our study demonstrated that the modified Atema, Kim HY, and our proposed scores were effective in predicting complicated appendicitis with high AUC and reasonable sensitivities. These scores have the potential to aid in the safe selection of patients for nonoperative management. However, further validation is required in larger, multicenter studies with a diverse patient population. Recent publications have shown that artificial neural networks may play a crucial role in this regard [20, 22]. Additionally, it is important to note that a prospective trial [23] focused on this issue is currently ongoing, and its results are eagerly awaited to further guide clinical decision-making.

### Supplementary Information

**Additional file 1: Supplementary Material 1.** Definitions of CT findings. **Supplementary Material 2.** Eight scoring systems under investigation. **Supplementary Material 3.** Diagnostic performance of current scores to predict complicated appendicitis based on the internal validation (n = 65).

## Data Availability

The data used to support the findings of this study are included in the article.

## Abbreviations

AUC: Area under the ROC curve
CT: Computed tomography
PACS: Picture Archiving and Communication System
ROC: Receiving Operator Characteristics

## References

[1] Di Saverio S, Podda M, De Simone B, Ceresoli M, Augustin G, Gori A (2020). Diagnosis and treatment of acute appendicitis: 2020 update of the WSES Jerusalem guidelines. World J Emerg Surg.

[2] Moris D, Paulson EK, Pappas TN (2021). Diagnosis and management of acute appendicitis in adults: a review. JAMA.

[3] Bom WJ, Scheijmans JCG, Salminen P, Boermeester MA (2021). Diagnosis of uncomplicated and complicated appendicitis in adults. Scand J Surg.

[4] Song H, Lee S, Park JH, Kim HY, Min HD, Jeon JJ (2021). Can patient triaging with clinical scoring systems reduce CT use in adolescents and young adults suspected of having appendicitis?. Radiology.

[5] Atema JJ, van Rossem CC, Leeuwenburgh MM, Stoker J, Boermeester MA (2015). Scoring system to distinguish uncomplicated from complicated acute appendicitis. Br J Surg.

[6] Kim TH, Cho BS, Jung JH, Lee MS, Jang JH, Kim CN (2015). Predictive factors to distinguish between patients with noncomplicated appendicitis and those with complicated appendicitis. Ann Coloproctol.

[7] Imaoka Y, Itamoto T, Takakura Y, Suzuki T, Ikeda S, Urushihara T (2016). Validity of predictive factors of acute complicated appendicitis. World J Emerg Surg.

[8] Avanesov M, Wiese NJ, Karul M, Guerreiro H, Keller S, Busch P (2018). Diagnostic prediction of complicated appendicitis by combined clinical and radiological appendicitis severity index (APSI). Eur Radiol.

[9] Khan MS, Siddiqui MTH, Shahzad N, Haider A, Chaudhry MBH, Alvi R (2019). Factors associated with complicated appendicitis: view from a low-middle income country. Cureus.

[10] Lin HA, Tsai HW, Chao CC, Lin SF (2021). Periappendiceal fat-stranding models for discriminating between complicated and uncomplicated acute appendicitis: a diagnostic and validation study. World J Emerg Surg.

[11] Kim HY, Park JH, Lee SS, Jeon JJ, Yoon CJ, Lee KH (2021). Differentiation between complicated and uncomplicated appendicitis: diagnostic model development and validation study. Abdom Radiol (NY).

[12] Geerdink TH, Augustinus S, Atema JJ, Jensch S, Vrouenraets BC, de Castro SMM (2021). Validation of a scoring system to distinguish uncomplicated from complicated appendicitis. J Surg Res.

[13] Fujiwara K, Abe A, Masatsugu T, Hirano T, Hiraka K, Sada M (2021). Usefulness of several factors and clinical scoring models in preoperative diagnosis of complicated appendicitis. PLoS One.

[14] Iamwat J, Teerasamit W, Apisarnthanarak P, Noppakunsomboon N, Kaewlai R (2021). Predictive ability of CT findings in the differentiation of complicated and uncomplicated appendicitis: a retrospective investigation of 201 patients undergone appendectomy at initial admission. Insights Imaging.

[15] Bröker MEE, van Lieshout EMM, van der Elst M, Stassen LPS, Schepers T (2012). Discriminating between simple and perforated appendicitis. J Surg Res.

[16] Chambers AC, Bismohun SL, Davies H, White P, Patil AV (2015). Predictive value of abnormally raised serum bilirubin in acute appendicitis: a cohort study. Int J Surg.

[17] Eddama MMR, Fragkos KC, Renshaw S, Aldridge M, Bough G, Bonthala L (2019). Logistic regression model to predict acute uncomplicated and complicated appendicitis. Ann Roy Coll Surg Engl.

[18] García-Amador C, Arteaga Peralta V, de la Plaza LR, Torralba M, Medina Velasco A, Ramia JM (2021). Evaluation of preoperative clinical and serological determinantions in complicated acute appendicitis: a score for predicting complicated appendicitis. Cir Esp.

[19] Mahankali SK, Ahamed SA, Gupta GSP, Razek AAKA (2021). CT based acute appendicitis severity index for acute appendicitis and validate its effectiveness in predicting complicated appendicitis. Emerg Radiol.

[20] Lin HA, Lin LT, Lin SF (2023). Application of artificial neural network models to differentiate between complicated and uncomplicated acute appendicitis. J Med Sys.

[21] Podda M, Andersson R, Boermeester M, Coccolini F, Sartelli M, Moore EE (2021). Do young patients with high clinical suspicion of appendicitis really need cross-sectional imaging? Proceedings from a highly controversial debate among the experts’ panel of 2020 WSES Jerusalem guidelines. J Trauma Acute Care Surg.

[22] Liang D, Fan Y, Zeng Y et al (2023) Development and validation of a deep learning and radiomics combined model for differentiating complicated from uncomplicated acute appendicitis. Available from: https://ssm.com/abstract=4297061. [Cited 2023 May 12] 10.1016/j.acra.2023.08.018 37775450

[23] Bom WJ, Scheijmans JCG, Ubels S, van Geloven AAW, Gans SL, Tytgat KMAJ (2022). Optimising diagnostics to discriminate complicated from uncomplicated appendicitis: a prospective cohort study protocol. BMJ Open.

