# Supplementary material for: Validation of scoring systems for the prediction of complicated appendicitis in adults using clinical and computed tomographic findings
Source: Insights Imaging. 2023 Nov 16;14:191. doi: 10.1186/s13244-023-01540-4 (PMC10654319; doi:10.1186/s13244-023-01540-4)
Supplement: Supplementary file 1 — Additional file 1: Supplementary Material 1. Definitions of CT findings. Supplementary Material 2. Eight scoring systems under investigation. Supplementary Material 3. Diagnostic performance of current scores to predict complicated appendicitis based on the internal validation (n = 65). [file 13244_2023_1540_MOESM1_ESM.docx]

**Validation of scoring systems for the prediction of complicated appendicitis in adults using clinical and computed tomographic findings**

**ELECTRONIC SUPPLEMENTARY MATERIAL**

**Supplementary Table 1: Definitions of CT findings**

| **CT findings** | **Definitions** |
| --- | --- |
| Appendix diameter | Outer-to-outer wall shortest diameter of appendix measured in the axial CT images that were perpendicular to the appendix’s axis. |
| Appendicolith | A hyperattenuating focus with a diameter >2 mm located either inside the appendiceal lumen or outside in fluid or fluid collection, shown on an unenhanced phase |
| Obstructive appendicolith | Appendicolith with dilation of the upstream appendix diameter greater than that of the appendicolith itself |
| Location of appendicolith | The location was described as being in the proximal 1/3, middle 1/3, or distal 1/3 of the appendix based on its position relative to the length of the appendix. |
| Periappendiceal fat stranding | Increased attenuation of fat surrounding appendix of >2 mm in thickness |
| Periappendiceal fluid | Extraluminal fluid around the appendix without encapsulation |
| Ascites | Free fluid, which is considered larger than a physiologic amount |
| Extraluminal air | Extraluminal air around the appendix or elsewhere in the abdomen and pelvis presumed to be related to appendicitis |

**Supplementary Material 2: Eight scoring systems under investigation.**

| **Scoring systems (year of publication)** | **Factors** | **Points** | **Total Points and cutoffs** | **Score performance** |
| --- | --- | --- | --- | --- |
| **Atema score (2015)** | Age >45 years | 2 | Total = 22 points  Cutoff (original) >6  Cutoff (this study; without CRP) >5 | *Original*: Se 97%, Sp 46%  *Validations*:  Geerdink et al; Se 90%, Sp 51%.  Fujiwara et al; Se 90%, Sp 83%, Ac 85%  Lin HA et al (2021; cutoff >7); Sp 77%, Sp 75%, AUC 0.83  Lin HA et al (2023); Se 64%, Sp 95% |
|  | Body temperature (^0^C)  <37.0  37.1-37.9  >38.0 | 0  2  4 |  |  |
|  | Duration of symptoms >48 hours | 2 |  |  |
|  | WBC count >13 × 10^9^/L | 2 |  |  |
|  | C-reactive protein (mg/L)*  <50  51-100  >100 | 0  2  3 |  |  |
|  | Extraluminal free air | 5 |  |  |
|  | Periappendiceal fluid | 2 |  |  |
|  | Appendicolith | 2 |  |  |
| **Imaoka score (2015)** | Body temperature >37.4 | 1 | Total = 3 points  Cutoff (original) >2  Cutoff (this study; without CRP) >1 | *Original:* Se 81% (cutoff >2), 100% (cutoff >1)  *Validations*:  Fujiwara et al (cutoff >1); Se 98%, Sp 48%, Ac 60%  Lin HA et al (2021; cutoff >1); Se 85%, Sp 69%, AUC 0.80  Lin HA et al (2023); Se 64%, Sp 97% |
|  | C-reactive protein >4.7 mg/dL* | 1 |  |  |
|  | Fluid collection around appendix | 1 |  |  |
| **Kim score (2015)** | C-reactive protein >5 mg/dL* | 1 | Total = 4 points  Cutoff (original) >2  Cutoff (this study; without CRP) >1 | *Original*: Se 89%, Sp 94%  *Validations*:  Lin HA et al (2021); Se 56%, Sp 86%, AUC 0.78  Lin HA et al (2023); Se 71%, Sp 95% |
|  | Appendiceal maximal diameter >10 mm | 1 |  |  |
|  | Ascites | 1 |  |  |
|  | Periappendiceal fat infiltration | 1 |  |  |
| **Avanesov score (2018)** | Age >52 years | 1 | Total = 10 points  Cutoff (original) = 4  Cutoff (this study) = 2 | *Original*: Se 82%, Sp 93%, Ac 87%, AUC 0.92  *Validations*:  Lin HA et al (cutoff >2); Se 81%, Sp 69%, AUC 0.81  Lin HA et al (2023); Se 79%, Sp 95%, AUC 0.95 |
|  | Temperature >37.5 ^0^C | 1 |  |  |
|  | Duration of symptoms >48 hours | 1 |  |  |
|  | Appendiceal maximal diameter >14 mm | 1 |  |  |
|  | Extraluminal free air | 1 |  |  |
|  | Periappendiceal fluid | 2 |  |  |
|  | Abscess | 3 |  |  |
| **Khan score (2019)** | Age (years)  40-59  >60 | 1  2 | Total = 4 points  Cutoff (original) = 2  Cutoff (this study) = 2 | *Validations*:  Lin HA et al (2021); Se 73, Sp 59, AUC 0.69  Lin HA et al (2023); Se 7%, Sp 99% |
|  | Duration of symptoms >48 hours | 1 |  |  |
|  | Appendicolith | 1 |  |  |
| **Lin score (Model 1; 2021)** | C-reactive protein (mg/dL)*  3.0-5.9  >6.0 | 2  3 | Total = 10 points  Cutoff (original) = 6  Cutoff (this study; without CRP) = 4 | *Original*: Se 83%, Sp 83%, AUC 0.88 |
|  | Periappendiceal fat stranding**  Grade 1  Grade 2  Grade 3 | 3  4  5 |  |  |
|  | Ascites | 1 |  |  |
|  | Appendicolith | 1 |  |  |
| **Lin score (Model 2; 2021)** | C-reactive protein (mg/dL)*  3.0-5.9  >6.0 | 2  3 | Total = 10 points  Cutoff (original) = 6  Cutoff (this study; without CRP) = 4 | *Original*: Se 81%, Sp 82%, AUC 0.88  *Validations*:  Lin HA et al (2023); Se 86%, Sp 92%, AUC 0.95 |
|  | Neutrophil-to-lymphocyte ratio >10 | 1 |  |  |
|  | Periappendiceal fat stranding**  Grade 1  Grade 2  Grade 3 | 3  4  5 |  |  |
|  | Ascites | 1 |  |  |
| **Kim HY score (2021)** | % Neutrophil >81% | 1 | Total = 6 points  Cutoff (original) = 3  Cutoff (this study) = 3 | *Original*: Se 93%, Sp 28%, AUC 0.81  *Validations*:  Lin HA et al (2021); Se 64%, Sp 88%, AUC 0.84  Lin HA et al (2023); Se 64%, Sp 100%, AUC 0.92 |
|  | Appendiceal maximal diameter >10 mm | 1 |  |  |
|  | Contrast enhancement wall defect | 1 |  |  |
|  | Extraluminal free air | 1 |  |  |
|  | Periappendiceal fat stranding | 1 |  |  |
|  | Abscess | 1 |  |  |

CRP = C-reactive protein

* This factor was removed in the modified versions.

**For the purpose of this investigation, grade 1, 2 and 3 were considered as having fat stranding.

**Supplementary Material 3: Diagnostic performance of current scores to predict complicated appendicitis based on the internal validation (n = 65)**

|  | **Current score^1^** | **Current score^2^** |
| --- | --- | --- |
| True positive | 37 | 35 |
| False positive | 15 | 11 |
| False negative | 0 | 6 |
| True negative | 13 | 13 |
| AUC | 0.844 (0.732 – 0.922) | 0.826 (0.712 – 0.909) |
| Sensitivity (%) | 100 (90.6 – 100) | 85.4 (71.6 – 93.1) |
| Specificity (%) | 46.4 (29.5 – 64.2) | 54.2 (35.1 – 72.1) |
| Positive predictive value (%) | 71.2 (57.7 – 81.7) | 76.1 (62.1 – 86.1) |
| Negative predictive value (%) | 100 (77.2 – 100) | 68.4 (46.0 – 84.6) |
| Accuracy (%) | 76.9 (65.4 – 85.5) | 73.9 (62.0 – 83.0) |

^1^using odds ratio

^2^using coefficient

Values in brackets represent 95% confidence interval
